# Zebrafish Functional Genomics

**DOI:** 10.1002/1097-0061(20000930)17:3<232::AID-YEA32>3.0.CO;2-B

**Published:** 2000

## Abstract

Artemis Pharmaceuticals have just announced a second saturation mutagenesis screen of the zebrafish, *Danio rerio*. In this interview with Stefan Schulte-Merker, who is Head of Fish Genetics at Artemis, we find out more about the purpose of the second screen and contrast it with the first screen which was carried out in Christaine Nüsslein-Volhard laboratory and which is now called ‘Tübingen One’.

*Stefan Schulte-Merker* has been working with zebrafish for over ten years and was involved in a number of genetic screens. He was also part of a team that produced a first-generation map of the ‘Goodfellow panel’ of radiation hybrids, which was intended to help in the cloning of the mutated genes. He is currently Head of Fish Genetics at Artemis Pharmaceuticals, in Tübingen, who are in the process of a second-round genetic screen, called ‘Tübingen 2000’.


**Yeast**
*Yeast* 2000; **17**: 232–234.

Interview: Stefan Schulte-Merker

### Zebrafish functional genomics

**Artemis Pharmaceuticals have just announced a second saturation mutagenesis screen of the zebrafish, *Danio rerio*. In this interview with Stefan Schulte-Merker, who is Head of Fish Genetics at Artemis, we find out more about the purpose of the second screen and contrast it with the first screen which was carried out in Christaine Nüsslein-Volhard laboratory and which is now called 'Tübingen One'.**

Stefan Schulte-Merker **has been working with zebrafish for over ten years and was involved in a number of genetic screens. He was also part of a team that produced a first-generation map of the 'Goodfellow panel' of radiation hybrids, which was intended to help in the cloning of the mutated genes. He is currently Head of Fish Genetics at Artemis Pharmaceuticals, in Tübingen, who are in the process of a second-round genetic screen, called 'Tübingen 2000'.** **Copyright © 2000 John Wiley & Sons, Ltd.**

***CFG: How many genes of developmental importance were identified in the first screen?***

SS-M: It is hard to say how many genes the first screen has uncovered. This would require mapping of all of the mutated genes, or checking of all mutants for complementation. As an estimate, we could use the fact that 4264 mutants were identified in Tu I (Tübingen 1, the first screen), with an allele frequency of 2.4. Therefore, one could argue that 1777 genes were identified (4264/2.4). However, the number 2.4 stems from 894 mutants and might therefore not be quite accurate.

***CFG: Did you see any bias in the types of genes you identified, are they predominantly genes of known or unknown function?***

SS-M: Only a few of the genes have been cloned so far, so it is hard to generalize about what types of genes have been found, or the representation of various pathways. So far, there is a strong bias towards known genes, since candidate cloning has, to date, been the primary method used to obtain the genes. However, increasingly, groups are using positional cloning and more novel genes are being identified. For example, the Zon lab recently found a novel iron transporter gene by positional cloning (Donovan et al., [2000]).

***CFG: By what criteria did you decide that the first screen was not saturating?***

SS-M: The trivial answer here is that you only find what you are looking for. The first screen was designed to uncover mutants affecting structures that one could identify by looking at the live embryos (with one exception, where fixed embryos were processed for staining retinotectal projections). If you do not screen for, say, osteogenesis or blood vessel formation, then you will not identify the genes controlling those processes. So, whenever you want to find the essential genes in a process that previously no-one has looked at, you basically have to do a new screen. Given the complexity of the early embryo and larva, there are many more genes to be found. Even for those pathways studied, the first screen has most likely not achieved saturation, as can be deduced from the isolation of genes involved in developmental pathways studies in Tu I, but not detected by the screen, and from independent projects by other groups. Also, there are several genes that have been found in the Driever/Fishman screens and not in the Tübingen screen and vice versa, indicating a lack of complete coverage in either screen.

***CFG: Do you have an estimate of how many of these genes remain to be found?***

SS-M: No, but it will be more than 1000 and probably less than 5000. However, the number depends on how you define genes that are important for early embryogenesis. One indication could be taken from the allele frequency of 2.4 that we observe, in experiments with *C. elegans* and *Drosophila*, it has been shown that an allele

Copyright © 2000 John Wiley & Sons, Ltd.

frequency of between 4 and 5 is required to achieve saturation, so we can infer that we probably missed our target by a factor of 2.

***CFG: Can you give some examples of the types/roles of genes you found in the first screen? Are the genes you found highly conserved across other species?***

SS-M: The number of types of mutants from the first screen is very high, and the only good way to get an impression is to look at Development 123: 1–481. All mutants are described there. One group of mutants that has been characterized particularly well is mutants affecting dorsoventral patterning in the early embryo. Here, many of the mutants have been cloned, and it turns out that genes within the BMP pathway have been found (BMP2 and BMP7, tolloid, chordino). These genes, and what's more important their function, have been conserved though evolution. Another example is the group of mutants affecting haematopoiesis. Here, genes have been identified that were known from humans (globin, NRAMP2, ALAS), but there were also genes identified that scientists knew had to exist, but which had not been uncovered. They were identified through cloning mutant genes that came out of the screen.

***CFG: What are the major differences between the two screens? For example, how will the phenotypic screens differ?***

SS-M: We have learned lessons from the first screen, even though it has to be said that there were no major shortcomings in the first screen. During Tu I, everybody screened for all phenotypes, while during Tu 2000, specialists will screen for only one particular (or two at most) trait(s). This way, it is easier to compare phenotypes (all heart phenotypes are seen by the same person), and the experience factor is higher, which means that subtle phenotypes will also be detected. The second screen will cover more genomes than the first.

The screens being done now have mostly a completely different focus. The first screen was used to look for genes involved in early patterning of the embryo, whilst the second is aiming more at later stages of organogenesis. Angiogenesis, chondrogenesis and osteogenesis are areas that have not been covered in previous screens. The assays we are using to look at these developmental pathways are more complex, requiring staining of vasculature, cartilage or bone prior to assessment of the fish. By contrast, in the first screen, with the exception of the staining for assay of the retinotectal projections, all the assays involved more straightforward observation of living embryos under the microscope. We did consider other avenues, such as the use of transgenic lines expressing genes that mark out tissue types, but we are limited in the types of screens we can apply; for example, the assays we choose must be robust and high throughput.

We are also trying to ensure that there will be at least some ability to look for multiple phenotypes per gene. We are using a database to log results, which should help to detect any correlations between phenotypes.

***CFG: Chemical mutagenesis is a random process and each fish may harbour several mutations. Do you plan to include steps to ensure that each phenotype is caused by mutation in only one gene?***

SS-M: Even though every fish harbours many different mutations, it is easy to decide whether you have a single hit or a double mutant because in the former case 1/4 of the embryos display the phenotype, while in double mutants only 1/16 gives a phenotype. With the genome being spread over 25 chromosomes, it is easy to separate the mutations. A possible problem could only come from linked double mutants; however, the likelihood of two neighbouring (linked) genes being mutated in the same genome is very small and can be neglected.

In conclusion, I would like to say that I feel that the future of zebrafish genomics lies in this type of genetic screen and the identification of the underlying genes. This remains the most amenable and rapid way of obtaining gene function and can also be modified to take in advances such as transgenic lines with marked tissue types. I would also argue that the zebrafish is still the most efficient vertebrate system for this approach.

### References

Donovan A, Brownlie A, Zhou Y, *et al.* 2000. Positional cloning of zebrafish ferroportin 1 identifies a conserved vertebrate iron exporter. *Nature* **403**: 776–781.

Copyright © 2000 John Wiley & Sons, Ltd. 

The Interview articles of Comparative and Functional Genomics aim to present a commentary on topical issues in genomics studies. They are a personal critical analysis, by the interviewee, of current work in their field of expertise, and aim at providing implications for future genomics studies.
This interview was undertaken by the Managing Editor, Dr Joanne Wixon.

**Dr Stefan Schulte-Merker**
Artemis Pharmaceuticals GmbH, Spemannstrasse 35. D-72076 Tübingen, Germany.

**http://www.artemis-pharmaceuticals.de/html/topic2000.html**

*The*
**Genomics**
*Website at Wiley*

**www.wiley.co.uk/genomics**

The **Genomics** website at Wiley is a new and **DYNAMIC** resource for the genomics community, offering **FREE** special feature articles and new information **EACH MONTH**.

Find out more about *Comparative and Functional Genomics*, and how to view all articles published this year **FREE OF CHARGE**!

Visit the **Library** for hot books in Genomics, Bioinformatics, Molecular Genetics and more.

Click on **Primary Research** for information on all our up-to-the minute journals, including: *Genesis*, *Bioessays*, *Gene Function and Disease*, and the *Journal of Gene Medicine*.

Let the **Genomics** website at Wiley be your **guide** to genomics-related web sites, manufacturers and suppliers, and a calendar of conferences.

WILEY

1516

Copyright © 2000 John Wiley & Sons, Ltd. 
